# Supplementary figures and images for: A Monte Carlo simulation study of sample size requirements for the Graded Response Model
Source: PLoS One. 2026 Apr 22;21(4):e0347684. doi: 10.1371/journal.pone.0347684 (PMC13102218; doi:10.1371/journal.pone.0347684)

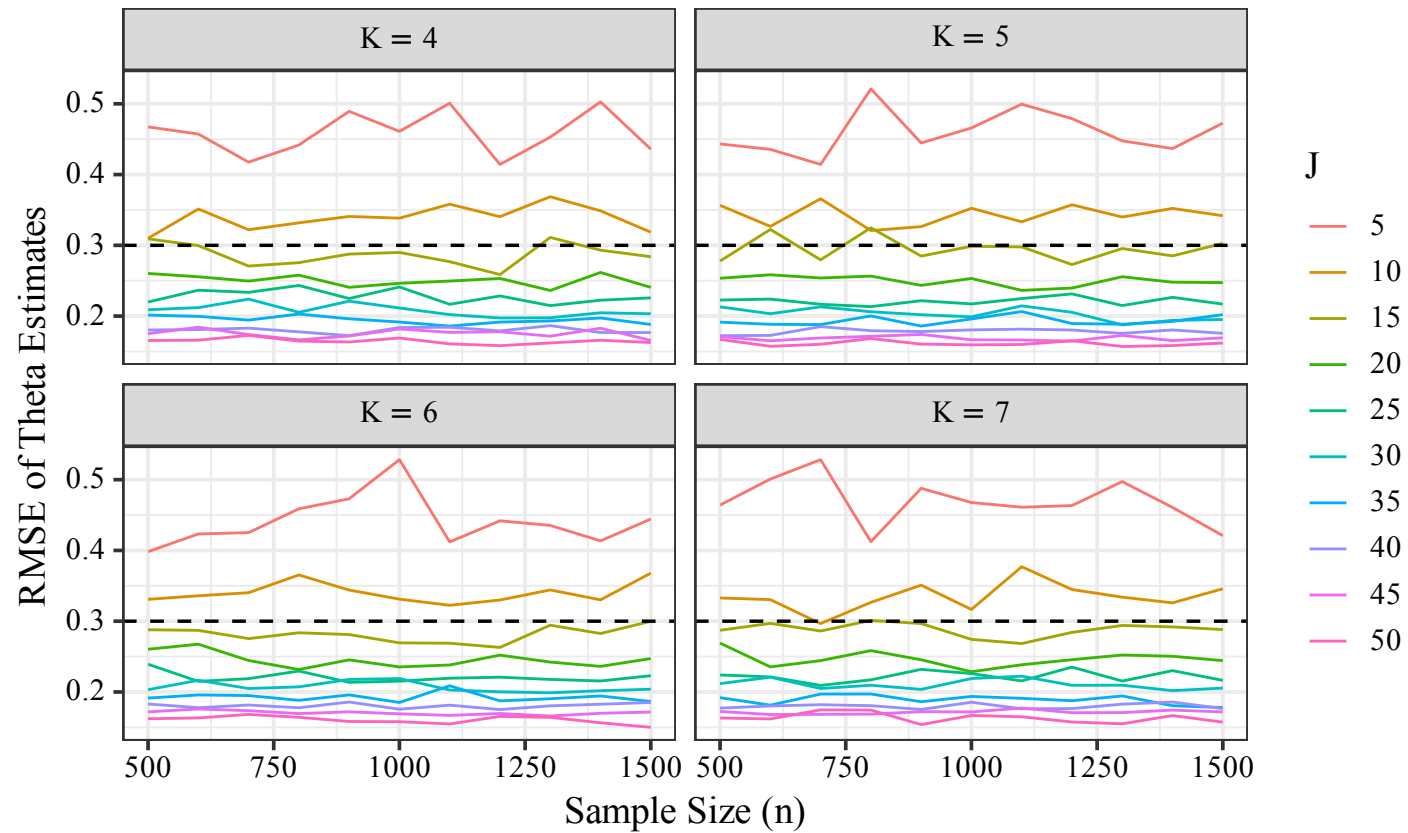

Supplement: S1 Fig — There is little difference from Fig. 7 using FPC-corrected RMSE. (PDF) [file pone.0347684.s001.pdf]

RMSE of Theta Estimates

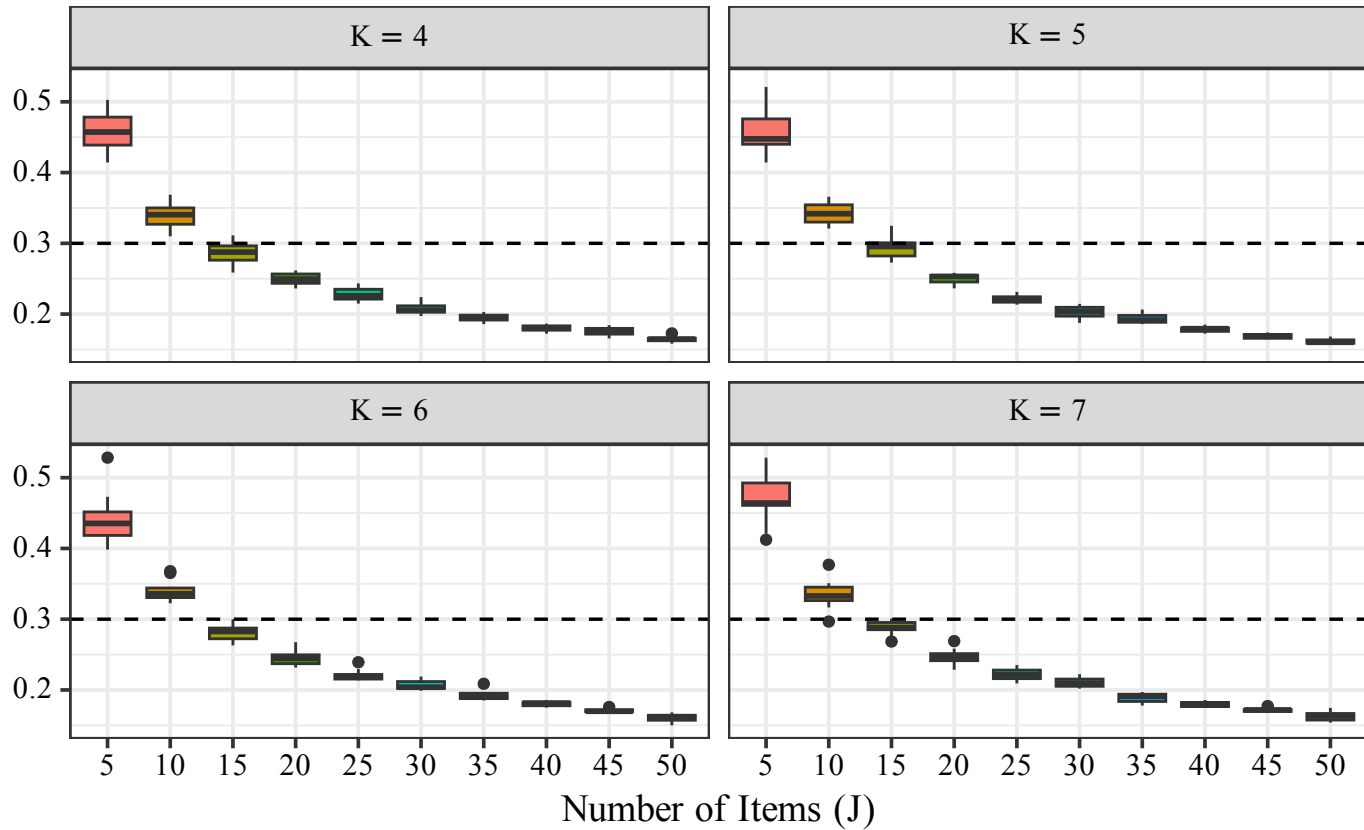

Supplement: S2 Fig — There is little difference from Fig. 8 using FPC-corrected RMSE. (PDF) [file pone.0347684.s002.pdf]
